# Gut colonization of *Bacteroides plebeius* suppresses colitis-associated colon cancer development

**DOI:** 10.1128/spectrum.02599-24

**Published:** 2025-01-13

**Authors:** Hung-Lin Chen, Po-Yuan Hu, Chang-Shan Chen, Wei-Han Lin, Daniel K. Hsu, Fu-Tong Liu, Tzu-Ching Meng

**Affiliations:** 1 Institute of Biomedical Sciences, Academia Sinica 71563 , Taipei City, Taiwan; 2 Institute of Biological Chemistry, Academia Sinica 71561 , Taipei City, Taiwan; 3 Master Program in Clinical Genomics and Proteomics, Taipei Medical University 38032 , Taipei City, Taiwan; 4 Institute of Biochemical Sciences, National Taiwan University 33561 , Taipei City, Taiwan; 5 Department of Dermatology, School of Medicine, University of California-Davis, Sacramento, California, USA; 6 Department of Dermatology, Keck School of Medicine USC, University of Southern California 12223 , Los Angeles, California, USA; Lerner Research Institute, Cleveland, Ohio, USA

**Keywords:** colon cancer, probiotic, gut microbiota, metabolites, colitis, *Bacteroides plebeius*

## Abstract

Colon cancer development may be initiated by multiple factors, including chronic inflammation, genetic disposition, and gut dysbiosis. The loss of beneficial bacteria and increased abundance of detrimental microbes exacerbates disease progression. *Bacteroides plebeius* (*B. plebeius*) is a human gut microbe, and its colon colonization is enhanced by a seaweed-supplemented diet. We found that mice orally administered with *B. plebeius* and fed a diet containing 1% seaweed developed a unique gut microbial composition. By linear discriminant analysis effect size analysis, we found that *B. plebeius* colonization increased the abundance of *Blautia coccoides* and reduced the abundance of *Akkermansia* sp. and *Dubosiella* sp. We also showed that colonization of *B. plebeius* suppressed the colon tumor development induced by azoxymethane/dextran sulfate sodium in specific-pathogen-free mice, coinciding with a reduced abundance of *Muribaculaceae* sp., *Closteridale* sp., and *Bilophila* sp. Moreover, *B. plebeius* colonization in gnotobiotic mice resulted in enhanced production of selected metabolites, including propionic, taurocholic, cholic, alpha-, and beta-muricholic, as well as ursodeoxycholic acids. Importantly, some of these metabolites show anti-inflammatory and tumor-suppressive effects. We conclude that *B. plebeius* is able to restructure the gut microbial community and produce beneficial metabolites, leading to inhibition of colitis-associated colon cancer development.

**IMPORTANCE:**

This work delves into the pivotal role of gut microbiota in suppressing the progression of colitis-associated colon cancer. By investigating the impact of *Bacteroides plebeius* that can be colonized in mouse gut by feeding the animal with seaweed diet, we unveil a novel mechanism through which this beneficial bacterium reshapes the gut microbial community and produces metabolites with anti-inflammatory and tumor-suppressive properties. Such findings underscore the potential of harnessing specific microbes, like *B. plebeius* shown in this study, to modulate the gut ecosystem and mitigate the risk of colitis-associated colon cancer.

## INTRODUCTION

Colorectal cancer (CRC) is the third most prevalent human cancer and the third most common cause of cancer-related death worldwide (1). The development of colon cancer has been reported to be associated with chronic inflammation in the gut (2). Mechanistically, CRC is a multifactorial and multi-step process. Immune cells and immune mediators, including cytokines and chemokines, as well as the activation of receptor-mediated signaling by epigenetic and/or genetic alterations in gut epithelial cells, initiate CRC development (3). Several gut microbes having immunomodulatory functions promote or suppress tumor cell proliferation (4); therefore, dysbiosis has been implicated in colon tumor development (4–6).

Some bacteria have been identified to be carcinogenic; these include *Fusobacterium nucleatum* (7, 8)*, Porphyromonas gingivalis* (9), enterotoxigenic *Bacteroides fragilis* (10), and polyketide synthase-positive (pks+) *Escherichia coli* (11). In promoting CRC development, in general, these pathogenic bacteria are equipped with virulent factors such as cell surface ligands or toxins that can interact with epithelial cell receptors. Such interactions perturb cell signaling homeostasis, resulting in uncontrolled host cell proliferation, deterioration of colon epithelial barrier function, and triggering of tissue inflammation through chemokine production, leading to bacterial translocation from the gut lumen to other tissues and severe immune cell infiltration (12, 13). In addition, some bacteria may grow on tumor cell surfaces and produce metabolites that inhibit tumor-suppressive immune cells (8) or activate tumor-promoting immune cells (14). Therefore, gut microbes may be involved in the development and progression of colon cancer.

The azoxymethane (AOM) and dextran sulfate sodium (DSS) mouse model (AOM/DSS hereafter) is commonly used to investigate the molecular pathogenesis of colitis-associated colon cancer. In this model, the tumor burden and number are suppressed by antibiotic treatment (15, 16), suggesting that bacterial community structure is critical for colitis-associated carcinogenesis. The involvement of the gut microbiota in regulating AOM/DSS-induced colon cancer may be explained by the presence of bacterial metabolites. For example, using this mouse model, it has been shown that some bacteria from the phylum *Firmicutes* can produce short-chain fatty acids (SCFAs), such as butyrate, which promote colonic cell signaling through the engagement of G protein-coupled receptors (e.g., GPR43 and GPR109) to suppress tumor development (17). These microbes use dietary fibers, which are not digested by host enzymes, to facilitate colonization and efficient production of metabolites.

To date, several bacteria have been reported to promote colon homeostasis and suppress colon cancer development (18–26). Among those bacteria not having been characterized in this regard, *Bacteroides plebeius*, which is also called *Phocaeicola plebeius* (27), is of particular interest. This bacterium is commonly found in Japanese people and has the ability to consume unique polysaccharides and arabinogalactan proteins in the seaweed (28), and its colonization and abundance in the gut of mice can be tuned through dietary supplementation with seaweed (29). Thus, it is feasible to evaluate its long-term effect on colon cancer development in experimental animals.

It has been suggested that maintenance of microbial composition throughout the entire period of AOM/DSS-induced inflammation is key to suppressing tumorigenesis (30). In this regard, a robust approach to maintaining specific microbes using prebiotics may be a solution to overcome the difficulty of probiotic colonization. Among potential probiotics that may be tuned by prebiotics, *B. plebeius* is of particular interest as it has acquired genes, including those from marine bacteria, encoding complex carbohydrate degradation enzymes (31). Such unique genetic features suggest that *Porphyra* spp. (seaweed) are important nutrients in controlling the colonization and propagation of *B. plebeius* in the host intestine. Indeed, a seaweed-supplemented diet not only facilitates *B. plebeius* colonization but also enhances its relative abundance in the mouse gut (28, 32). However, whether the long-term propagation of *B. plebeius* exerts any effect on the inhibition of colon cancer development is heretofore unknown.

In this study, we aimed to investigate the role of diet-regulated colonization of *B. plebeius* in AOM/DSS-induced mouse models of colitis-associated tumor. We used a seaweed diet to facilitate the long-term colonization of *B. plebeius* in gnotobiotic and specific-pathogen-free (SPF) mice. We then characterized the metabolites produced by *B. plebeius* after successful colonization. Consequently, we demonstrated that *B. plebeius* inhibits colon cancer development and elucidated the underlying mechanism by which *B. plebeius* exerts its effect on tumor suppression.

## RESULTS

### *B. plebeius* colonization in gnotobiotic mice modulates colon tissue mRNA profiles

**Fig 1:**
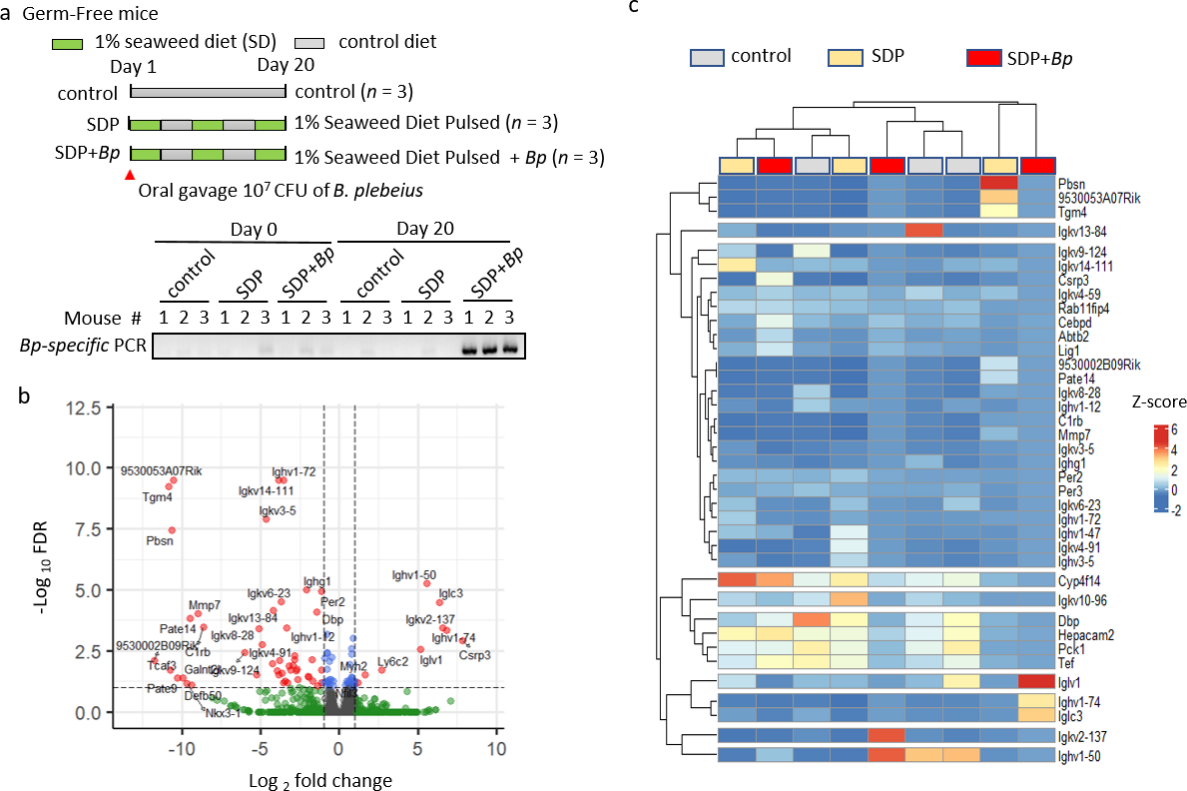
RNA-seq analyses of the effect of seaweed diet + *B. plebeius* colonization on the colon tissue of gnotobiotic mice. (**a**) Schematic diagram of *B. plebeius* administration and seaweed diet intervention in germ-free mice. *B. plebeius* was administered orally by gavage once on day 1, and a seaweed diet was provided at 4-day intervals, interspersed with control diet. *B. plebeius*-specific PCR of fecal DNA was performed to validate the colonization of *B. plebeius*. Day 0 represents no treatment control. SDP: 1% seaweed diet pulsed. (**b**) RNA-seq analysis was performed using colon tissue samples from the control (*n* = 3), seaweed diet (*n* = 3), and seaweed diet + *B. plebeius* (*n* = 3) groups. Volcano scatterplot shows log2FC vs -log FDR comparing seaweed diet (*n* = 3) and seaweed diet + *B. plebeius* (*n* = 3) groups. Gene symbols with FDR *P* < 0.01 and log2FC > 1 are annotated on the graph. (**c**) Heatmap analysis of differentially expressed genes (FDR *P* < 0.01 and log2FC > 1, comparing seaweed diet and seaweed diet + *B. plebeius* groups) was performed using transcripts per million (TPM) values from individual mice in the control, seaweed diet, and seaweed diet + *B. plebeius* groups. Hierarchical clustering of gene expression was performed using the ComplexHeatmap R package software, and *Z*-scores are presented by colors in the heatmap.

To examine the effect of *B. plebeius* colonization on colon cells in gnotobiotic mice, we first perform oral gavage of *B. plebeius* with or without seaweed diet supplementation and collect colon tissue for RNAseq analysis at day 20. In the control groups, fecal samples from gnotobiotic C57BL/6JN mice with and without seaweed diet supplementation did not contain *B. plebeius*, as determined by *B. plebeius*-specific PCR (Fig. 1a). On the other hand, in mice supplemented with seaweed and orally inoculated with *B. plebeius*, we found that *B. plebeius* was colonized successfully in the gut (Fig. 1a). We analyzed mRNA expression in mouse colon tissues using RNA-Seq analysis. The volcano plot shows the log2 fold change (log2FC) vs log false discovery rate (-log FDR) of mRNA expression between the seaweed diet and seaweed diet + *B. plebeius* groups (Fig. 1b). Thirty-eight genes were differentially expressed in these groups, with FDR *P* < 0.01, and are annotated in Fig. 1b. In addition, we examined the mRNA levels of these 38 genes in the colon tissues of individual mice using heatmap analysis and performed Pearson’s correlation clustering analysis to make genes with a similar expression pattern cluster together (Fig. 1c). We identified many differentially expressed genes between the seaweed diet and seaweed diet + *B. plebeius* groups. CCAAT enhancer-binding protein delta (*Cebpd*), ankyrin repeat and Broad-Complex, Tramtrack and Bric brac (BTB) domain containing 2 (*Abtb2*), and DNA ligase 1 (*Lig1*) mRNA were clustered together and consistently upregulated in the seaweed diet + *B. plebeius* group mice (Fig. 1c). However, none of the identified differentially expressed genes are known to be associated with the inflammatory response, suggesting that *B. plebeius* colonization alone does not elicit such a response.

### Seaweed diet-mediated *B. plebeius* colonization increases microbial diversity and establishes a unique gut microbiome in SPF mice

**Fig 2:**
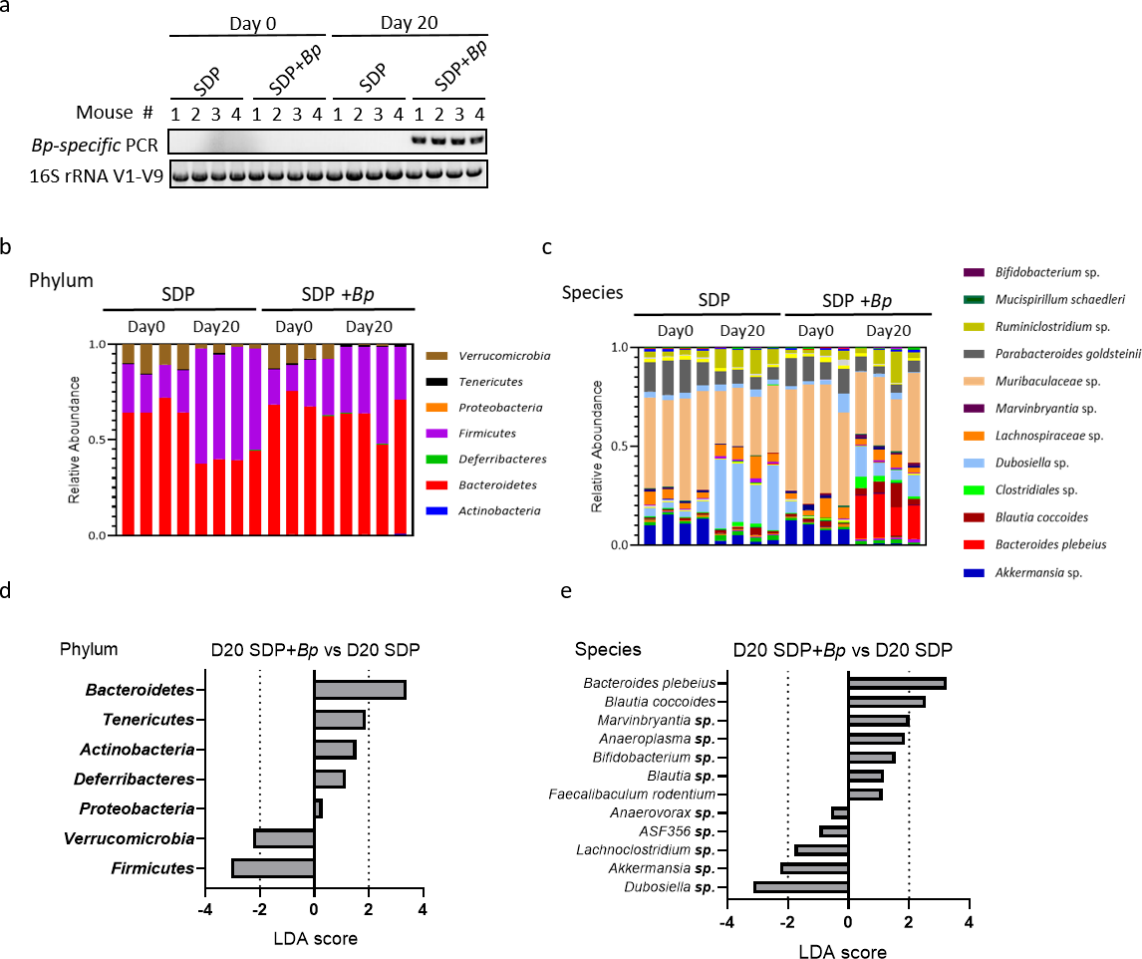
Gut microbiota composition analyses of SPF mice with seaweed diet and *B. plebeius* colonization. (**a**) *B. plebeius-*specific PCR of fecal DNA was performed to validate the colonization of *B. plebeius*. The 16S full-length PCR (V1–V9) of fecal DNA samples was used as a control. (**b and c**) Microbial composition in the seaweed diet (*n* = 4) and seaweed diet + *B. plebeius* (*n* = 4) groups on day 0 (before any treatment) and day 20 at the phylum and species levels. The microbiota analysis was performed using the loop-genomics technology, and the full-length 16S rRNA genes were sequenced. Only the top 12 abundant species are annotated in the legend. (**d and e**) LEfSe was used to compare the microbial composition of the seaweed diet and seaweed diet + *B. plebeius* groups. The relative abundance of all phyla is shown; only species with a *P*-value of less than 0.05 are shown.

Next, we orally administered *B. plebeius* in SPF C57BL/6JN mice. Using *B. plebeius*-specific PCR and 16S rRNA full-length sequencing, we found that *B. plebeius* was not present in the fecal samples of indigenous SPF mice (Fig. 2a). On the other hand, *B. plebeius* was colonized in SPF mice by oral gavage when the administration was followed by a seaweed diet fed at 4-day intervals for a total of three times in 20 days, interspersed with control diet (the same experimental procedure used for colonization in germ-free mice; Fig. 1a). At the phylum level, the seaweed diet increased the abundance of *Firmicutes*; however, the seaweed diet + *B. plebeius* group did not show this effect, and *Firmicutes* levels were unchanged in three out of four mice in the group (Fig. 2b). On day 20, the relative abundance of *B. plebeius* increased to 30% in the seaweed diet + *B. plebeius* group (Fig. 2c). Comparison of the gut microbial composition in the seaweed diet and the seaweed diet +*B. plebeius* groups using linear discriminant analysis (LDA) effect size (LEfSe) revealed that at the phylum level, the abundance of *Bacteroidetes* (LDA score > 2) was higher and that of *Firmicutes* (LDA score < −2) was lower in the seaweed diet + *B. plebeius* group (Fig. 2d). At the species level, apart from *B. plebeius*, *Blautia coccoides* was enriched and abundances of *Akkermansia* sp. and *Dubosiella* sp. decreased, in the seaweed diet + *B. plebeius* group (Fig. 2e). These results suggest that a seaweed diet can be used to facilitate *B. plebeius* predominance in the gut microbial community of SPF mice.

### Seaweed diet*-*mediated *B. plebeius* colonization suppresses AOM/DSS-induced colon carcinogenesis in SPF mice

**Fig 3:**
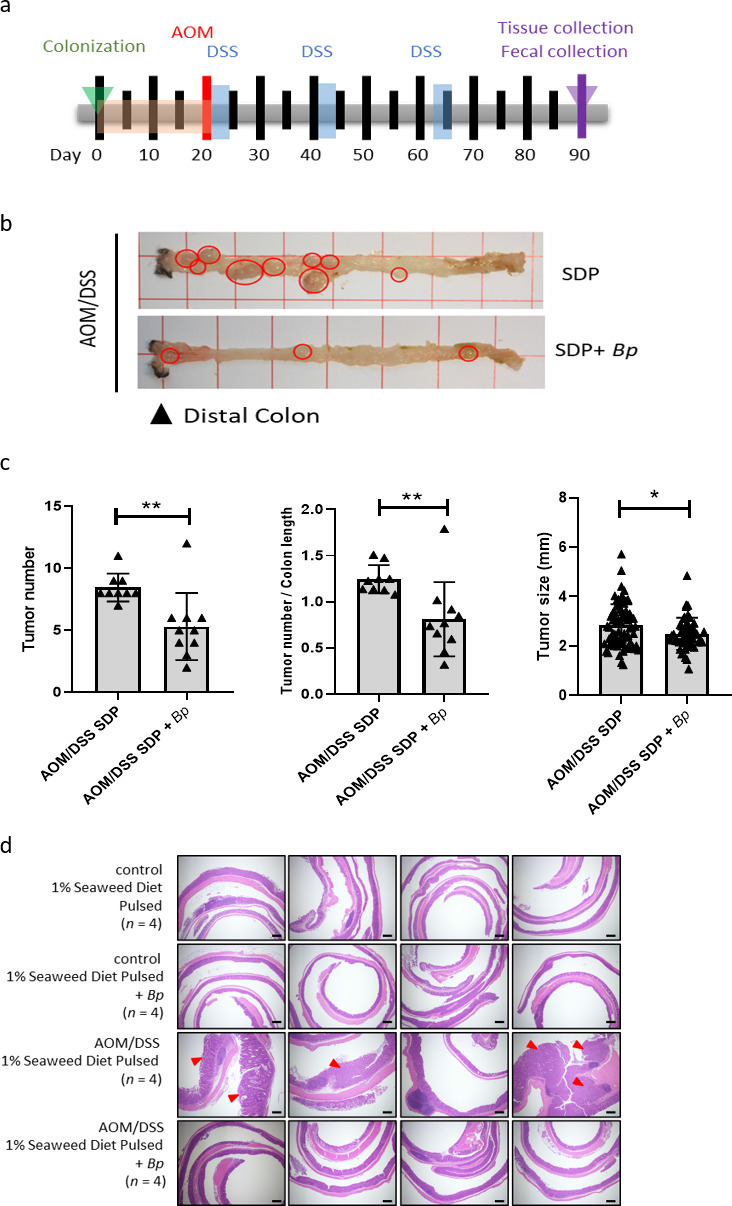
Seaweed diet and *B. plebeius* suppress AOM/DSS-induced colon carcinogenesis in SPF mice. (**a**) Schematic timeline of seaweed diet and *B. plebeius* colonization followed by AOM/DSS-induced colitis-associated CRC development in SPF mice. Three cycles of DSS treatments were performed followed by AOM intraperitoneal injection. (**b**) Colon tissues from AOM/DSS-treated SPF mice of the seaweed diet (*n* = 9) and seaweed diet + *B. plebeius* (*n* = 10) groups. A representative colon tissue is presented from each of the two groups. The distal colon is oriented to the left as indicated in the picture. (**c**) Tumor number in AOM/DSS-treated SPF mice of the seaweed diet (*n* = 9) and seaweed diet + *B. plebeius* (*n* = 10) groups was counted from individual mouse, and tumor burden represents the number of tumors divided by the length of the colon. The tumor size is an aggregate of all individual tumors from all different mice. (**d**) Hematoxylin and eosin staining of Swiss-rolled colon sections from mice from the four groups, namely, the control seaweed diet (*n* = 4), control seaweed diet + *B. plebeius* (*n* = 4), AOM/DSS-treated seaweed diet (*n* = 4), and AOM/DSS-treated seaweed diet + *B. plebeius* (*n* = 4) groups. Scale bar: 200 µm. *P < 0.05 and **P < 0.01.

Alterations in the gut microbial composition can modulate colon cancer development. To examine whether *B. plebeius* colonization can regulate colon cancer development in mouse model, we first administered *B. plebeius* to SPF mice by oral gavage and then fed the mice a seaweed diet at 4-day intervals for a total of three times in 20 days, interspersed with control diet. This was followed by one peritoneal AOM injection and three cycles of oral DSS treatment (Fig. 3a) to induced colon cancer formation. The colon tissues were collected at the end of the AOM/DSS treatment at day 90 for the following analysis. Our results showed that not only the tumor number and tumor burden but also the tumor size (Fig. 3b and c) in the colon of AOM/DSS-treated mice fed the seaweed diet plus *B. plebeius* were significantly reduced compared to those in mice fed the seaweed diet alone. Representative images of colon tissues from each of these two groups, as well as controls without AOM/DSS treatment, are presented in Fig. 3b.

After 10 weeks of AOM/DSS treatment for colon cancer development, we performed hematoxylin and eosin (H&E) staining of Swiss-rolled colon sections from four mice in each group (control- and AOM/DSS-treated mice fed the seaweed diet with and without *B. plebeius*). H&E staining of colon tissue indicated significant lymphoid aggregates and adenoma development after AOM/DSS treatment compared to the controls (Fig. 3d). Importantly, histological assessment indicated that the severity of hyperplasia was lower in distal and middle colon of AOM/DSS-treated mice given the seaweed diet plus *B. plebeius*, compared with AOM/DSS-treated mice with seaweed diet alone. However, there was no significant difference in the colon length and histological colitis scores between mice with and without *B. plebeius* colonization after AOM/DSS treatment (Fig. S1).

### Genes associated with cytokine-cytokine receptor interactions are downregulated in the colon of the seaweed + *B. plebeius*-administrated SPF mice

**Fig 4:**
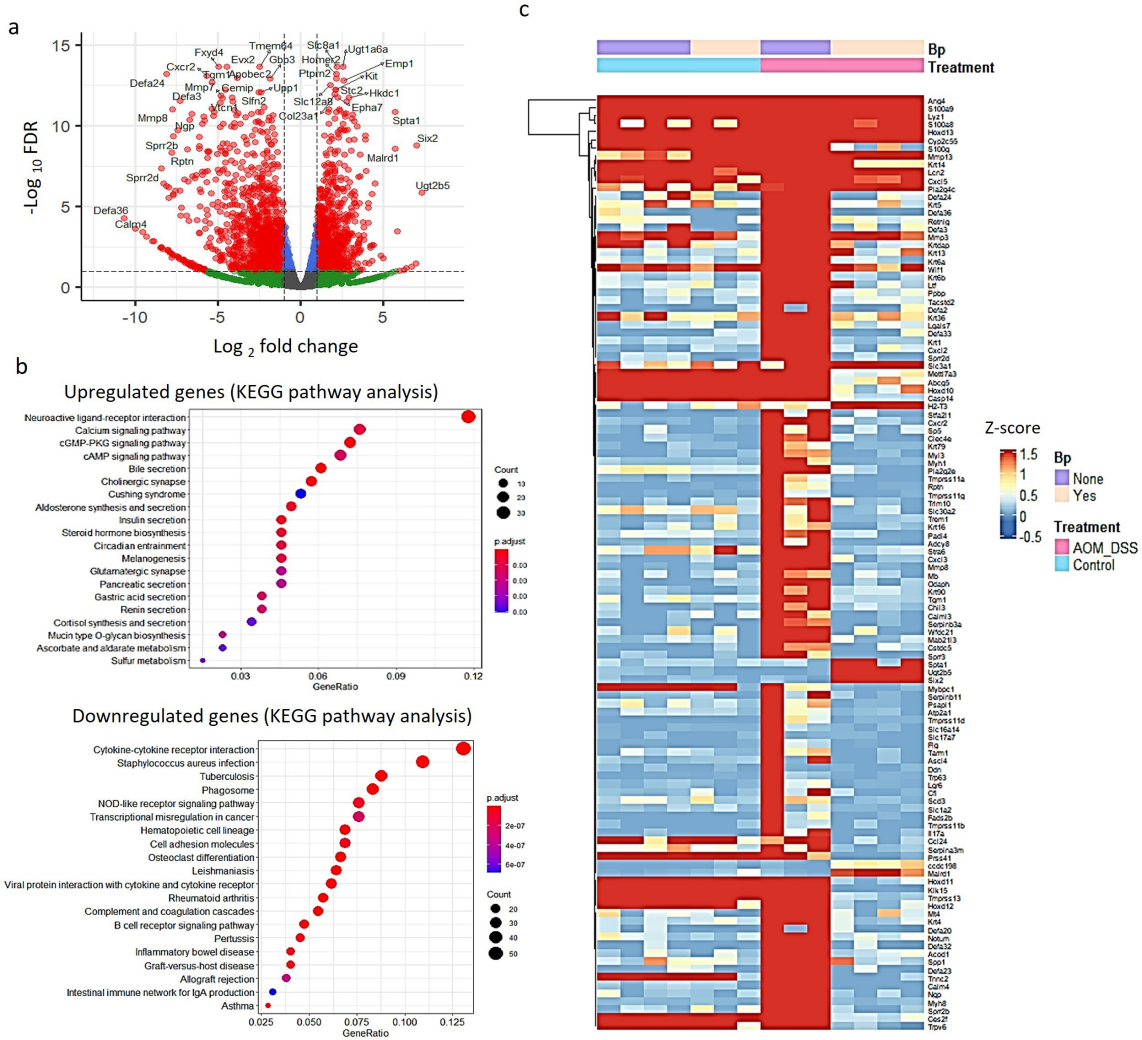
RNA-seq analyses of colon tissue samples in SPF mice. (**a**) Volcano scatter plot comparing the AOM/DSS with seaweed diet (*n* = 3) and seaweed diet + *B. plebeius* (*n* = 4) groups showing log2FC vs -log FDR is presented. Genes with FDR *P* < 0.01 and log2FC > 2 were designated as differentially expressed genes for pathway analysis. Only partial gene symbols are annotated on the graph. (**b**) KEGG and GO pathway analyses were performed using the clusterProfiler package in R software. The differentially expressed upregulated and downregulated genes were selected as described in (**a**). (**c**) Heatmap analysis of differentially expressed genes (FDR *P* < 0.01 and log2FC > 5, comparing AOM/DSS with seaweed diet and seaweed diet + *B. plebeius* groups) was performed using transcripts per million (TPM) values from individual mice of the control with seaweed diet (*n* = 4), control with seaweed diet + *B. plebeius* (*n* = 3), AOM/DSS with seaweed diet (*n* = 3), and AOM/DSS with seaweed diet + *B. plebeius* (*n* = 4) groups. Hierarchical clustering of gene expression profiles was performed using the ComplexHeatmap R package, and *Z*-scores are presented by colors in the heatmap.

To explore the mechanism by which seaweed diet plus *B. plebeius* suppresses AOM/DSS-induced carcinogenesis, we performed RNA-seq analysis using colon tissues collected at day 90 as described in Fig. 3a. There are four groups of SPF mice: (i) control seaweed diet, (ii) control seaweed diet + *B. plebeius*, (iii) AOM/DSS-treated seaweed diet, and (iv) AOM/DSS-treated seaweed diet + *B. plebeius* in our study (Fig. 3d). The mice oral gavage with *B. plebeius* (BP) without seaweed fail to colonized to colon by fecal BP-specific PCR analysis at day 20 after colonized, thus excluded for the analysis, which is consistent to previous study (29). We first examined the differentially expressed genes in AOM/DSS-treated mice fed the seaweed diet alone and those fed the seaweed diet + *B. plebeius*. As shown in the volcano plot (Fig. 4a), we identified 796 differentially expressed genes, among which 252 were upregulated and 544 downregulated with FDR *P* < 0.01 and log2FC > 2. These upregulated and downregulated genes were used for the Kyoto Encyclopedia of Genes and Genomes (KEGG) and gene ontology (GO) pathway enrichment analyses, respectively (Fig. 4b). We further sorted all the mapped pathways using *P*-values. The enriched KEGG pathways of the upregulated genes were identified, in which the top five pathways include those associated with neuroactive ligand-receptor interaction, calcium signaling, cyclic guanosine monophosphate (cGMP)-PKG signaling, bile secretion, and cholinergic synapse (Fig. 4b; Fig. S2). The top five KEGG pathways of the downregulated genes include those associated with cytokine-cytokine receptor interactions (Fig. S3), *Staphylococcus aureus* infection, tuberculosis, phagosomes, and nucleotide-binding oligomerization domain-like receptor signaling pathways. The top five enriched GO pathways of the upregulated genes include those associated with various channels, passive transmembrane transporter, and uridine diphosphate (UDP)-glycosyltransferase activity. The top five GO pathways of the downregulated genes include those associated with immune receptor activity, peptidase inhibitor activity, and endopeptidase inhibitor activity.

We performed a hierarchical clustering analysis of the differentially expressed genes (FDR *P* < 0.01 and log2FC > 5, comparing the AOM/DSS-treated seaweed diet and the seaweed diet + *B. plebeius* groups) in individual mice of all four groups mentioned above. The results shown in the heatmap (Fig. 4c) suggested that several genes were upregulated in the AOM/DSS-treated seaweed diet + *B. plebeius* group compared to the AOM/DSS-treated seaweed diet group, including spectrin alpha (*Spta1*), meprin-A5-protein tyrosin phosphatase mu (MAM) and low density lipoprotein (LDL) receptor class A domain containing 1 (*Malrd1*), sine oculis-related homeobox 2 (*Six2*), and UDP glucuronosyltransferase 2 family, polypeptide B5 (*Ugy2b5*). In contrast, several genes were downregulated in the AOM/DSS-treated seaweed diet + *B. plebeius* group compared to the AOM/DSS-treated seaweed diet group, including chemokine (C-X-C motif) receptor 2 (*Cxcr2*), C-type lectin domain family 4, member e (*Clec4e*), chemokine (C-X-C motif) ligand 3 (*Cxcl3*), galectin-7 (*Lgals7*), and matrix metallopeptidase 8 (*Mmp8*). The potential roles of these genes in colon cancer development are described in the section Discussion.

### Seaweed diet plus *B. plebeius* treatment in AOM/DSS-induced mice shapes the gut microbial community

**Fig 5:**
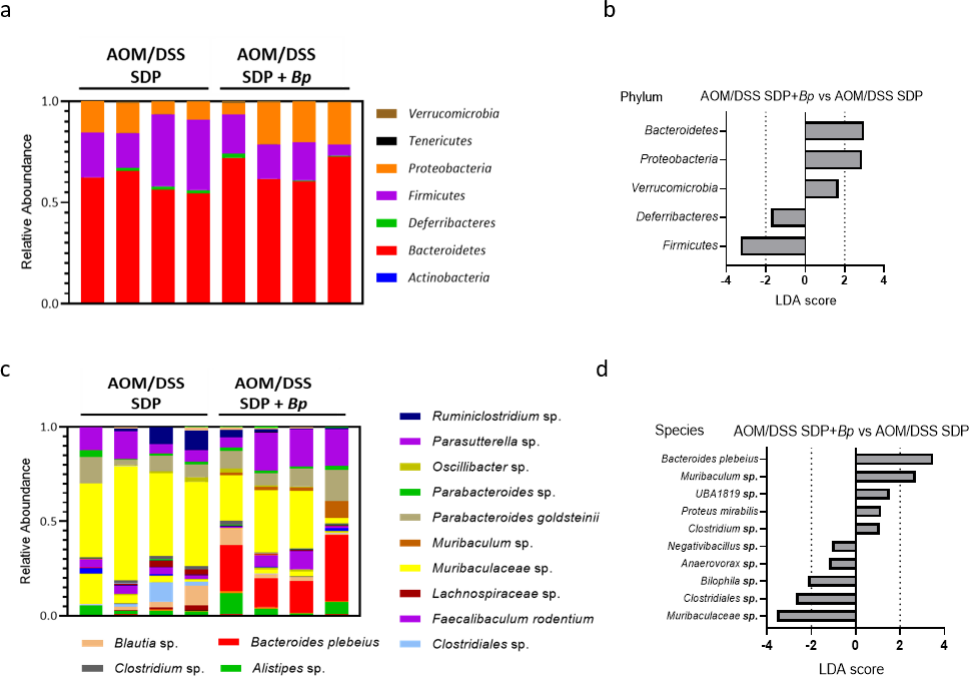
Gut microbial composition analyses of AOM/DSS-induced colitis-associated colon cancer in SPF mice with seaweed diet and *B. plebeius* colonization. (**a and b**) Microbiota composition in the gut of mice in the AOM/DSS with seaweed diet (*n* = 4) and seaweed diet + *B. plebeius* (*n* = 4) groups on day 100 at the phylum and species levels. The microbiota analysis was performed using loop-genomics technology where the full length of the 16S rRNA genes was sequenced. Only the top 14 abundant species are annotated in the legend. (**c and d**) LEfSe was used to compare the microbial composition of the seaweed diet and seaweed diet + *B. plebeius* groups. The relative abundances of all phyla are shown; only species with a *P*-value of less than 0.05 are shown.

To acquire higher-resolution microbiome classification, especially at the species level, we used Loop Genomics technology to examine samples. After microbial classification, we examined the alpha diversity of each biological sample using the Shannon diversity index. The richness and evenness of the gut microbial community decreased after AOM/DSS treatment; however, there was no significant difference in richness and evenness between the seaweed diet + *B. plebeius* and seaweed diet alone groups (Fig. S4a). Next, to measure the change in microbial communities between the aforementioned groups, beta diversity was calculated using the Bray-Curtis index. We found that each group of mice had a distinct community structure (Fig. S4b). Furthermore, we applied LEfSe to identify statistically significant biomarkers between the different groups. Among the AOM/DSS-treated mice, the abundance of *Proteobacteria* and *Bacteroidetes* increased, while that of *Firmicutes* decreased at the phylum level in the seaweed diet + *B. plebeius* group compared with the seaweed diet control (Fig. 5a and b). At the species level, *B. plebeius* and *Muribaculum* sp. were enriched in the seaweed diet + *B. plebeius* group compared to the seaweed diet group (LDA score > 2, *P* < 0.05). The abundances of *Bilophila* sp., *Clostridium* sp., and *Bilophila* sp. decreased with *B. plebeius* colonization (LDA score < −2, *P* < 0.05; Fig. 5c and d). It was shown that the abundance of some commensal microbes was altered in response to *B. plebeius* colonization in the mouse gut (32). Our study provides evidence that *B. plebeius* colonization in AOM/DSS-treated mice shapes the gut microbial community.

### *B. plebeius* colonization in gnotobiotic mice promotes the production of specific metabolites

**Fig 6:**
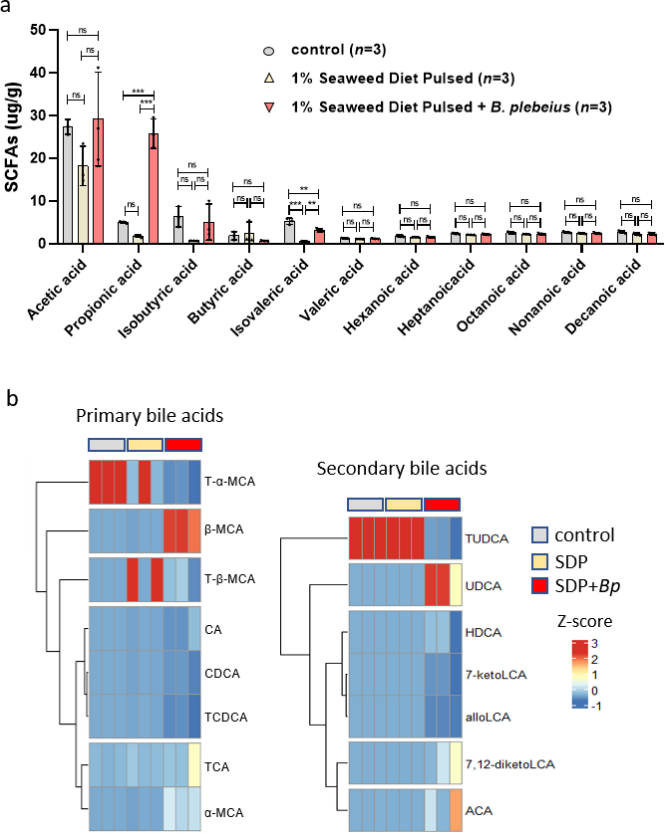
Fecal metabolite analyses of seaweed diet + *B. plebeius* colonization in gnotobiotic mice. (**a**) Fecal SCFA levels were quantified using GC-MS, and the levels of acetic, propionic, isobutyric, butyric, isovaleric, valeric, hexanoic, heptanoic, octanoic, and decanoic acids were calculated as microgram per gram of fecal samples. (**b**) Fecal bile acids were analyzed using ultra-high-performance liquid chromatography-parallel reaction monitoring with tandem mass spectrometry. Of the 41 targeted bile acids, 18 were identified. The 14 bile acids above the lower limit of quantification, i.e., 0.49 nmol/L, are presented in the heatmap. Hierarchical clustering of the bile acid levels was performed using the ComplexHeatmap R package, and *Z*-scores are presented by colors in the heatmap. **P < 0.01 and ***P < 0.001. ns: not significant.

To investigate the possible mechanism by which *B. plebeius* colonization reduces colon inflammation and tumor development in AOM/DSS-treated mice, we measured the metabolites produced by *B. plebeius* colonized in gnotobiotic mice fed a seaweed diet. SCFAs (33) and secondary bile acid metabolites (34) produced by gut bacteria are known to regulate colon inflammation and tumor growth. Fecal samples from the control, seaweed diet, and seaweed diet + *B. plebeius* groups on day 20 were collected, and SCFAs and bile acid levels were determined. Fecal SCFA levels were quantified using gas chromatography-mass spectrometry (GC-MS), and the data revealed that acetic, propionic, isobutyric, and isovaleric acids were the major SCFAs in gnotobiotic mice. The seaweed diet + *B. plebeius* group produced significantly higher amounts of propionic acid (25.75 ± 2.84 µg/g) than the control (5.05 ± 0.18 µg/g) and seaweed diet (1.780 ± 0.22 µg/g) groups (Fig. 6a). Fecal bile acids were analyzed using ultra-high-performance liquid chromatography coupled with tandem mass spectrometry. Eighteen bile acids were detected among the 41 targeted bile acids examined (Fig. 6b). Fourteen bile acids that were above the lower limits of quantification levels (0.49 nmol/L) are presented in the heatmap (Fig. 6b). Compared with the seaweed group, fecal samples isolated from the seaweed + *B. plebeius* group showed a lower level of tauro-α-muricholic acid (T-α-MCA), tauroursodeoxycholic acid, taurocholic acid, and taurochenodeoxycholic acid but a higher level of α-muricholic acid (α-MCA), β-muricholic acid (β-MCA), and ursodeoxycholic acid (UDCA; Fig. 6b). The concentrations of these bile acids are listed in Table S1. These results suggest that colonization of *B. plebeius* promotes the production of primary and secondary metabolites in germ-free mice. In addition, we treated gnotobiotic mice with a control bacterium along with the seaweed diet. In the case of *Escherichia coli* O19ab, we did not observe a colonization-induced effect on fecal propionic acid and UDCA (Fig. S5), which can be promoted by colonization of *B. plebeius* (Fig. 6B). It was reported that *B. plebeius* possesses bile acids metabolism genes, including bsh, baiG, and baiE (35). We thus performed *in vitro* analysis and found that mono *B. plebeius* culture supplemented with primary bile acid induced an increased level of second metabolites, including chenodeoxycholic acid (CDCA), LCA, and UDCA (Fig. S6). Taken together, our approach using diet-facilitated colonization in gnotobiotic mice and the analysis of *in vitro* mono culture have confirmed that *B. plebeius* promotes the production of specific metabolites, which may contribute to beneficial biological effects such as suppression of AOM/DSS-induced colon tumors (Fig. S7). However, *B. plebeius* colonization in SPF mice did not show a significant effect on the production of specific metabolites. The amounts of short-chain fatty acids and bile acid were comparable between the seaweed + *B. plebeius* group and seaweed group (Fig. S8).

## DISCUSSION

The development of colon cancer occurs over a long period. Our study validated that dietary supplementation with seaweed can modulate the sustained abundance of *B. plebeius*. The efficient modulation of gut microbes is a challenge in the treatment of diseases. Here, we demonstrated that *B. plebeius* colonization facilitated by a seaweed-supplemented diet can suppress AOM/DSS-induced colon cancer development. Tumor numbers, tumor sizes, and lymphoid aggregates were significantly reduced in *B. plebeius-*colonized mice. In addition, although AOM/DSS-treated SPF mice showed shorter colon lengths, *B. plebeius* colonization had no effect on AOM/DSS-affected colon length and colitis pathological score (Fig. S1). This may suggest that *B. plebeius* colonization suppresses colon cancer growth independent of colon inflammation induced by DSS. However, further analysis of fecal DNA and colon tissue RNA revealed that the high abundance of *B. plebeius* resulted in a unique microbial community structure in association with a reduced inflammatory cytokine expression after colon cancer development. We found that *B. plebeius* and the seaweed diet uniquely regulated the expression of genes following AOM/DSS treatment. Immune-related genes, such as chemokine (*Cxcr2*) (36), C-type lectin (*Clec4e*) (37), chemokine ligand (*Cxcl3*) (38), tumor growth-related genes, galectin-7 (*Lgals7*) (39), and matrix metallopeptidase 8 (*Mmp8*) (40), were downregulated by colonization of *B. plebeius* in colon tissues of the mice exposed to AOM/DSS (Fig. 4c). Insights into whether downregulation of these genes correlates with reduced colon cancer growth, and how such decrease of gene expression may synergistically control tumor development would be important for the development of new *B. plebeius*-based therapies, thus requiring further investigation.

We also found that *B. plebeius* colonization can upregulate *Spta1*, *Malrd1*, *Six2*, and *Ugy2b5* expression in SPF mice exposed to AOM/DSS (Fig. 4c). Although little is known about the role of these genes in tumor development, they may act as potential signatures for *B. plebeius* colonization and metabolite production. Additional investigations will unravel whether these genes are upregulated by *B. plebeius* alone or by its metabolites. Such new findings may help understand host signaling pathways and subsequent cellular function affected by *B. plebeius*.

We propose that diet-facilitated *B. plebeius* colonization may suppress colon cancer development by altering the bacterial community, thus increasing the relative abundance of bacteria with anti-inflammatory functions. Nonetheless, it would be interesting to test whether the presence of *B. plebeius* itself can execute an anti-tumor role. For this, we examined the effect of *B. plebeius* colonization in gnotobiotic mice treated with AOM/DSS and found that, compared to the control animals, the tumor number was reduced in the groups of seaweed diet + *B. plebeius* and *B. plebeius* alone (Fig. S8a and b). These results suggest that *B. plebeius* colonization alone, even without seaweed diet supplement, can suppress tumor development in this model.

*B. plebeius* has been shown to acquire genes encoding complex carbohydrate-degrading enzymes, including porphyranases and agarases*,* from marine bacteria (28). This may explain why seaweed promoted the colonization and propagation of *B. plebeius* in *vivo* in this study. In addition, *B. plebeius* possesses several carbohydrate-degrading enzymes including α-galactosidase, β-galactosidase, α-glucosidase, β-glucosidase, and α-fucosidase (41). In colon cancer, fucose, (sialyl)T, (sialyl)Tn, and Lewis X/A levels increase and promote tumor growth (42). The abundance of *B. plebeius* may degrade fucose and suppress tumor development *in vivo*. This is consistent with our results showing *B. plebeius* colonization in germ-free mice suppressed AOM/DSS-induced colon cancer (Fig. S8).

We showed that *B. plebeius* is sufficient to reduce AOM/DSS-induced colon cancer in gnotobiotic mice. However, it is difficult to conclude whether *B. plebeius* alone is sufficient for tumor suppression in the SPF mouse. It is well known that SPF mice and gnotobiotic mice show different immune system development (43) and susceptibility to AOM/DSS-induced tumor development (44). In this regard, we cannot rule out that *B. plebeius* manifests its effects on cancer growth via different mechanisms in SPF and germ-free mice. Nevertheless, our results suggest that metabolites produced by *B. plebeius* may contribute to its generic tumor suppression function. Identification of the key factors that control the antitumor ability of *B. plebeius* in SPF mice must be undertaken in the future.

Metabolites are critical bioproducts generated by gut microbes and facilitate host health. All *B. plebeius* strains grow in the presence of bile (41). Therefore, we characterized the changes in fecal metabolite composition, such as SCFAs and bile acids, in germ-free mice colonized with *B. plebeius JCM 12973*. Acetic acid was the most abundant SCFA identified in the feces of germ-free mice (Fig. 6a). However, in the seaweed diet + *B. plebeius* group, fecal propionic acid concentration was comparable to that of acetic acid. In addition to SCFAs, we observed that *B. plebeius* could metabolize the primary bile acid taurocholic acid and generate the secondary metabolite UDCA. It was noted that our germ-free mice had less β-muricholic acid production compared to that reported in previous publication (45). Further characterization of the diet and mouse genetic background should provide additional insight. In addition, *B. plebeius* can stimulate the production of specific primary bile acids, such as cholic acid, CDCA, α-MCA, and β-MCA. The level of CDCA has been reported to correlate with liver sinusoidal endothelial cell CXCL16 mRNA expression, which can promote CXCR6 + hepatic nature killer T (NKT) cell recruitment to the liver and inhibit liver tumor growth in a tumor metastasis model (46). Secondary bile acids such as deoxycholic acid are cytotoxic to epithelial cells and promote colon cell dysplasia (34). UDCA directly inhibits colon cancer development and cell growth (47) and is a known prescription medication for cholesterol gallstone dissolution and primary biliary cholangitis. We treated HT-29 human colorectal adenocarcinoma cells with UDCA and found that UDCA inhibited G protein-coupled receptor (GPCR) CXCR4 expression (Fig. S9). The CXCL12/CXCR4 axis is a critical target for inflammation-driven CRC progression, and high CXCR4 expression in patients with colon cancer increases the risk of recurrence and poor survival (48, 49). Thus, our results suggest that metabolites of *B. plebeius*, such as UDCA, may play an important role in suppressing inflammation and colon cancer development. Understanding the effect of bile acids on host gene expression in the context of inflammation warrants further investigation. Our results suggest that *B. plebeius* metabolites may contain active compounds that can activate GPCRs and may be developed as anticancer drugs (50). These active compounds can be identified using existing cell-based drug-screening platforms (51). Furthermore, the identification of other bacteria and the active metabolites they produce as antitumor agents will be instrumental in identifying new drugs for colon cancer treatment. In conclusion, the findings illustrated in this diagram suggest that *B. plebeius* can effectively restructure the gut microbial community and produce beneficial metabolites, leading to the inhibition of colitis-associated colon cancer development. This highlights the potential of *B. plebeius* as a therapeutic agent in cancer prevention, underscoring the intricate relationship between diet, gut microbiota, and colon cancer (Fig. S10).

## MATERIALS AND METHODS

### Gnotobiotic and SPF mice

Six- to 8-week-old male C57BL/6JN gnotobiotic mice bred and handled in the isolator of the National Laboratory Animal Center (NLAC) Taiwan were used. Six- to 8-week-old male C57BL/6JN SPF mice born and raised in two animal facilities, NLAC and Academia Sinica SPF Animal Facility (AS Core), were used to study AOM/DSS-induced tumor development. All procedures, including seaweed diet, *B. plebeius* oral administration, and establishment of the AOM/DSS colitis-induced carcinogenesis mouse model, were approved by the Animal Care Committee of Academia Sinica and NLAC (protocol ID: 20–02-1448 and NLAC-108-M-034-R1, respectively).

### Bacterial strain and anaerobic culture conditions

*B. plebeius* (type strain JCM 12973=M12= DSM17135) was purchased from the Japan Collection of Microorganisms. The authenticity of this bacterium was verified using whole-genome sequencing by integrating Illumina short reads and nanopore long-read sequencing. The assembled sequence and genome annotation map are listed on GitHub (https://github.com/hunglinchen2003/Bacteria-WGS-assembly). *B. plebeius* was cultured on an anaerobic blood agar plate and anaerobically cooked chopped meat medium (Creative, New Taipei City, Taiwan) in an anaerobic chamber (Whitley DG250 Workstation; cs Biotech, Taipei, Taiwan). The storage and revival conditions for *B. plebeius* were the same as previously described (32).

### *B. plebeius* oral gavage and seaweed diet treatment

To harvest live bacteria for oral administration, we centrifuged the 48 h bacterial culture at 8,000 *× g* for 5 min, followed by two washes with phosphate-buffered saline (PBS); the culture was adjusted to 10^8^ colony forming unit (CFU)/mL, and 100 µL of the bacteria (10^7^ CFU) was given to each mouse by oral gavage. The control mice were administered 100 µL PBS. On day 1, 10^7^ CFU of *B. plebeius* was administered by oral gavage. The seaweed diet group received a custom AIN-93 growing rodent diet containing 1% seaweed nori at the indicated times, as described in the experimental timeline in Fig. 1 to 3. Mice were fed a seaweed diet for 4 days, followed by a control diet (AIN-93-based diet). Raw organic Nori seaweed powder was obtained from *Porphyra yezoensis* (RawNori.com, USA). The animal diet was formulated and manufactured by Research Diet, Inc. (New Brunswick, NJ, USA).

### Fecal DNA extraction

Fecal samples were collected from animals 1–3 days before the initiation of the experimental protocol for DNA extraction and metabolite analysis. DNA was extracted from stool samples using the QIAamp Fast DNA Stool Mini Kit (QIAGEN, CA, USA). Briefly, stool samples (24–200 mg) were suspended in 200 µL of buffer with a steel bead and homogenized by strong vortexing for 1 min. The suspension was heated for 5 min at 95°C. After centrifugation at 10,000 × *g* for 10 min, 15 µL of proteinase K and 200 µL of buffer (provided by QIAamp Fast DNA stool mini kit) were added to the suspension, followed by incubation for 10 min at 70°C. Then, 200 µL ethanol (96%–100%) was added to 600 µL suspension, the mixture was applied to a spin column, and the filtrates were discarded. After washing with two buffers, the DNA in the spin column was eluted with 100 µL of buffer. The DNA concentration was measured using a NonoDrop2000 spectrophotometer (Thermo Fisher Scientific, San Jose, CA, USA).

### Sample preparation, library construction, and sequencing

Purified DNA was quantified using the Qubit dsDNA HS assay (Thermo Fisher Scientific, San Jose, CA, USA), and DNA integrity was examined using the Agilent 4200 Tape Station system (Agilent Technologies, Palo Alto, CA, USA). The LoopSeq 16S Long Reads 24-plex Kit (Loop Genomics, San Hose, CA, USA) was used to generate the full-length 16S rDNA amplicon library according to the manufacturer’s instructions in the user manual. The libraries were sequenced on an Illumina NovaSeq platform (Illumina, San Diego, CA, USA) using the paired-end 150 bp mode. Sample preparation, library construction, and sequencing were performed by Welgene Biotech Co. Ltd. (Taipei, Republic of Korea).

### Bacteria taxonomical and community analyses

The raw sequence data were assembled into contigs using Loop Genomics Technology and mapped to a custom SILVA database (52). Contig sequence processing and filtering were performed by Welgene Biotech Co., Ltd. (Taipei, ROC). Taxa abundance and rank abundance curves were calculated and plotted using Qiime2 (53). Alpha diversity indices (richness, Chao1, ACE, Shannon diversity, Simpson diversity, Inverse Simpson, Shannon evenness, Simpson evenness, and Good’s coverage) were calculated using the Mothur and MicrobiomeAnalyst tools (54). Rarefaction curves and species accumulation curves, as well as the beta diversity indices (Bray-Curtis matrix, principal coordinate analysis (PCoA), and non-metric multidimentional scalling (NMDS)), were calculated using the Phyloseq (55) and VEGAN3 R packages (37).

### Establishment of AOM and DSS mouse model of colon cancer

On day 0, mice first received *B. plebeius* or PBS control oral gavage and seaweed diet, as described above. On day 21, mice were intraperitoneally injected with 12 mg/kg of AOM (Sigma, Cat.A2853). On days 20, 39, and 57, the drinking water was supplemented with dextran sulfate sodium salt (DSS colitis grade, 36–50 kDa; MP Biomedicals; Cat. 216011080; 3% DSS for SPF mice) and (1% DSS for gnotobiotic mice) for 5 days. The body weight of each mouse was measured every alternate day from day 0. The fecal samples of mice were collected on days 0, 20, 39, and 57 before each DSS treatment and at the endpoint of each animal experiment. Those mice kept at the Academia Sinica SPF animal facility (AS core) were euthanized on day 100, and those SPF and gnotobiotic mice kept at NLAC were euthanized on day 90. Serum samples and colon tissues were collected; half of the tissues were used for the Swiss-rolled tissue paraffin embedding for H&E staining, while RNA from the other half of the tissues was extracted for RNA-seq analysis.

### Histology and pathological examination

Paraffin-embedded mouse colon sections were prepared and H&E stained at the Pathology Core Laboratory (IBMS, Academia Sinica, and National Laboratory Animal Center, Taiwan). The microscopic image of low-grade dysplasia in the colon tissue was similar to that of a human colonic adenoma. The severity of hyperplasia and intestinal neoplasms was diagnosed by two pathologists from NLAC according to the criteria described by Boivin et al. (56). Colitis was recorded and scored by two pathologists from NLAC according to the following morphological features: grade 0, normal colonic mucosa; grade 1, shortening and loss of the basal one-third of the crypts with mild inflammation and edema in the mucosa; grade 2, loss of the basal two-thirds of the crypts with moderate inflammation in the mucosa; grade 3, loss of all crypts with severe inflammation in the mucosa but with the surface epithelium remaining; and grade 4, loss of all crypts and the surface epithelium with severe inflammation in the mucosa, muscularis propria, and submucosa (57).

### RNA sequencing and data analysis

Total RNA was extracted from mouse colon tissues using TRIzol Reagent (Invitrogen, USA) according to the manufacturer’s instructions. Agilent’s SureSelect Strand-Specific RNA Library Preparation Kit was used for library construction, followed by AMPure XP beads (Beckman Coulter, USA). The sequence was determined using Illumina sequencing-by-synthesis technology (Illumina, USA). Illumina’s official base-calling program, bcl2fastq conversion software v2.20., was used to convert binary base call (BCL) files from all Illumina sequencing platforms into FASTQ reads. After the removal of adapter sequences, quality trimming was performed to remove low-quality reads/bases. Next, we used the CLC genomic workbench (QIAGEN, USA), an RNA analysis module, for sequence mapping to the reference mouse genome GRCm38, using default mapping parameters. Differentially expressed genes were determined using the CLC genomic workbench with fold change (FC) and the false discovery rate (FDR). The volcano plot shows the relationship between the *P*-values of a statistical test and FC among the samples. The log2FC values are plotted on the x-axis, and the log10 FDR is plotted on the y-axis. For GO and KEGG pathway analyses, differentially expressed genes were analyzed using the clusterProfiler package (58) in R software.

### Metabolite extraction

Fecal samples were weighed in a tube. After the addition of 1,000 µL of the extracting solvent (acetonitrile-methanol-water, 2:2:1, containing 0.1% formic acid and 50 nmol/L internal standards), the samples were vortexed for 30 s, homogenized at 35 Hz for 4 min, and sonicated for 5 min in an ice-water bath. The homogenate and sonication cycles were repeated three times, followed by incubation at −40°C for 1 h and centrifugation at 4°C for 15 min. The resulting supernatants were transferred to LC-MS vials and stored at −80°C until UHPLC-QE orbitrap/MS analysis.

### Ultra-high-performance liquid chromatography-parallel reaction monitoring-mass spectrometry analysis for bile acid quantification

Ultra-high-performance liquid chromatography (UHPLC) separation was carried out using an Agilent 1290 Infinity series UHPLC System (Agilent Technologies, USA) equipped with a Waters ACQUITY UPLC BEH C18 column (150 × 2.1 mm, 1.7 µm, Waters). Mobile phase A consisted of 1 mmol/L ammonium acetate and 1 mmol/L acetic acid in water, and mobile phase B consisted of acetonitrile. The column temperature was set at 60°C. The auto-sampler temperature was set at 4°C, and the injection volume was 1 µL. A Q Exactive Focus mass spectrometer (Thermo Fisher Scientific, MA, USA) was used for assay development. Typical ion source parameters were as follows: spray voltage = +3,500/–3,100 V, sheath gas (N2) flow rate = 40, auxiliary gas (N2) flow rate = 15, sweep gas (N2) flow rate = 0, auxiliary gas (N2) temperature = 350°C, and capillary temperature = 320°C.

The parallel reaction monitoring parameters for each of the targeted analytes were optimized by injecting standard solutions of individual analytes into the atmospheric pressure ionization (API) source of the mass spectrometer. Because most of the analytes did not show product ions that were acceptable for quantification, the precursor ion at high resolution was selected for quantification.

### Short-chain fatty acid analysis using GC-MS

GC-MS analysis was performed using an Agilent 7890 B gas chromatograph system coupled with an Agilent 5977 B mass spectrometer. The system used an HP-FFAP capillary column. A 1 µL aliquot of the analyte was injected in split mode (5:1). Helium was used as the carrier gas. The front inlet purge flow rate was 3 mL min^−1^, and the gas flow rate through the column was 1 mL min^−1^. The initial temperature was maintained at 80°C for 1 min, raised to 150°C at a rate of 5°C min^−1^, then held for 1 min, raised to 180°C at a rate of 10°C min^−1^, held for 0 min, raised to 245°C at a rate of 40°C min^−1^, and then held for 10 min. The injection, transfer line, quad, and ion source temperatures were 240°C, 240°C, 230°C, and 150°C, respectively. The energy was −70 eV in the electron impact mode. Mass spectrometry data were acquired in the Scan/SIM mode with an m/z range of 33–150 after a solvent delay of 2.5 min.

### Statistical analysis

Bacterial alpha and beta diversity were calculated using the R packages Phyloseq, Vegan, and MicrobiomeAnalystR (38). We performed an analysis of variance (ANOVA) on Shannon alpha diversity indices between groups and a permutational MANOVA (PERMANOVA) test for Bray-Curtis indices. The taxonomical profile and statistical test of pairs of samples were generated and calculated using LEfSe (59). The quantitative metabolite data are presented as the mean ± SEM, SE, or SD unless indicated otherwise. We performed one-way ANOVA with Tukey’s multiple-comparison test, as described in the figure legends, to compare the groups of samples. Differences with *P* values less than 0.05 were considered statistically significant.

## Data Availability

The sequence data of 16S rRNA full-length genes and RNA-Seq data of mouse colon tissues have been deposited in the NCBI SRA database under the BioProject accession PRJNA758419.

## SUPPLEMENTAL MATERIAL

Supplemental material spectrum.02599-24-s0001.docx Fig. S1 to S10; Table S1.

ASM does not own the copyrights to Supplemental Material that may be linked to, or accessed through, an article. The authors have granted ASM a non-exclusive, world-wide license to publish the Supplemental Material files. Please contact the corresponding author directly for reuse.
