# Supplementary material for: Gut colonization of Bacteroides plebeius suppresses colitis-associated colon cancer development
Source: Microbiol Spectr. 2025 Jan 13;13(2):e02599-24. doi: 10.1128/spectrum.02599-24 (PMC11792494; doi:10.1128/spectrum.02599-24)
Supplement: Supplemental material — Fig. S1 to S10; Table S1. [file spectrum.02599-24-s0001.docx]

**Supplementary information**

**Gut colonization of *Bacteroides plebeius* suppresses colitis-associated colon cancer development**

**Authors**

Hung-Lin Chen^1,2,3*^, Po-Yuan Hu^1^, Chang-Shan Chen^2,4^, Wei-Han Lin^1^, Daniel K. Hsu^5^, Fu-Tong Liu^1, 6*^and Tzu-Ching Meng^2,4*^

**Affiliations**

^1^Institute of Biomedical Sciences, Academia Sinica, Taipei, Taiwan

^2^Institute of Biological Chemistry, Academia Sinica, Taipei, Taiwan

^3^Master Program in Clinical Genomics and Proteomics, Taipei Medical University, Taipei, Taiwan

^4^Institute of Biochemical Sciences, National Taiwan University, Taipei, Taiwan

^5^Department of Dermatology, School of Medicine, University of California-Davis, Sacramento, CA, USA

^6^Department of Dermatology, Keck School of Medicine, University of Southern California, Los Angeles, CA 90033, USA

*Corresponding Authors


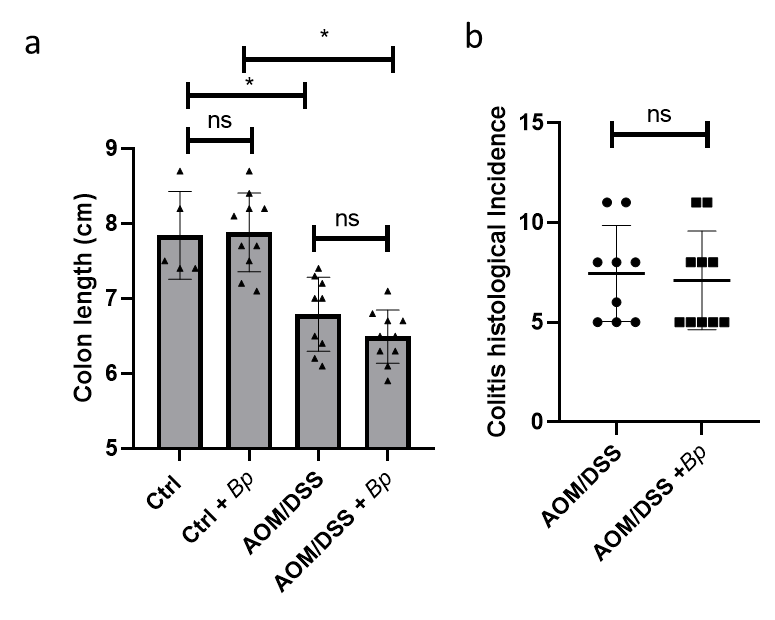


Supplementary Figure 1. The colon length abd colitis histological incidence were analysis using mice at day 90 of AOM/DSS treatment as descibed in Fig. 3a. (a) Colon length of mice that were fed with seaweed diet and treated with or without AOM/DSS and Bp. (b) Colitis histological incidence of mice orally gavaged with and without Bp, and all mice were fed with SDP and treated with AOM/DSS. *p < 0.5. ns: not significant.


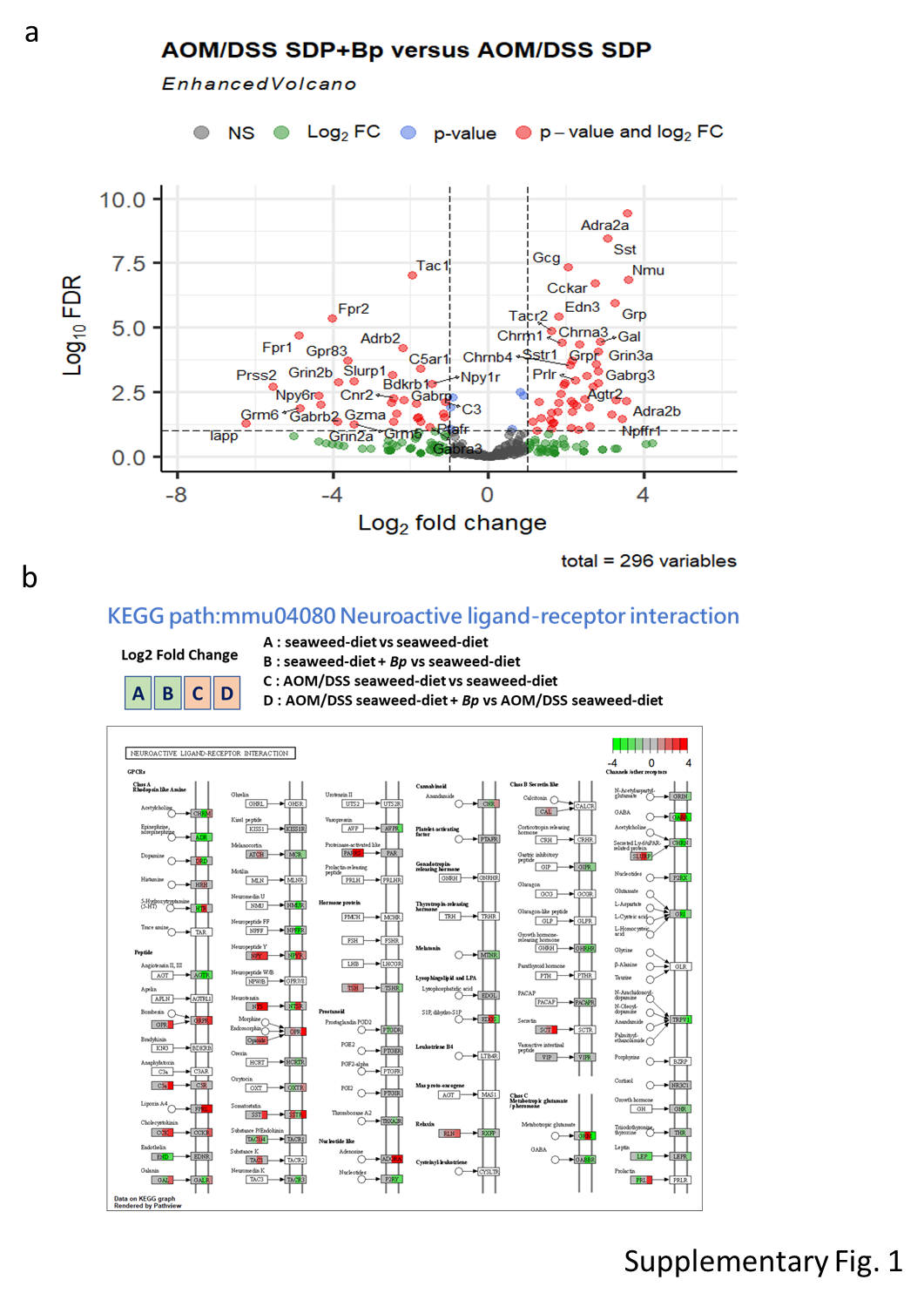


**Supplementary Figure 2.** Volcano plot (a) and KEGG pathway (b) analyses of neuroactive ligand-receptor interaction pathway (KEGG: mmu04080).


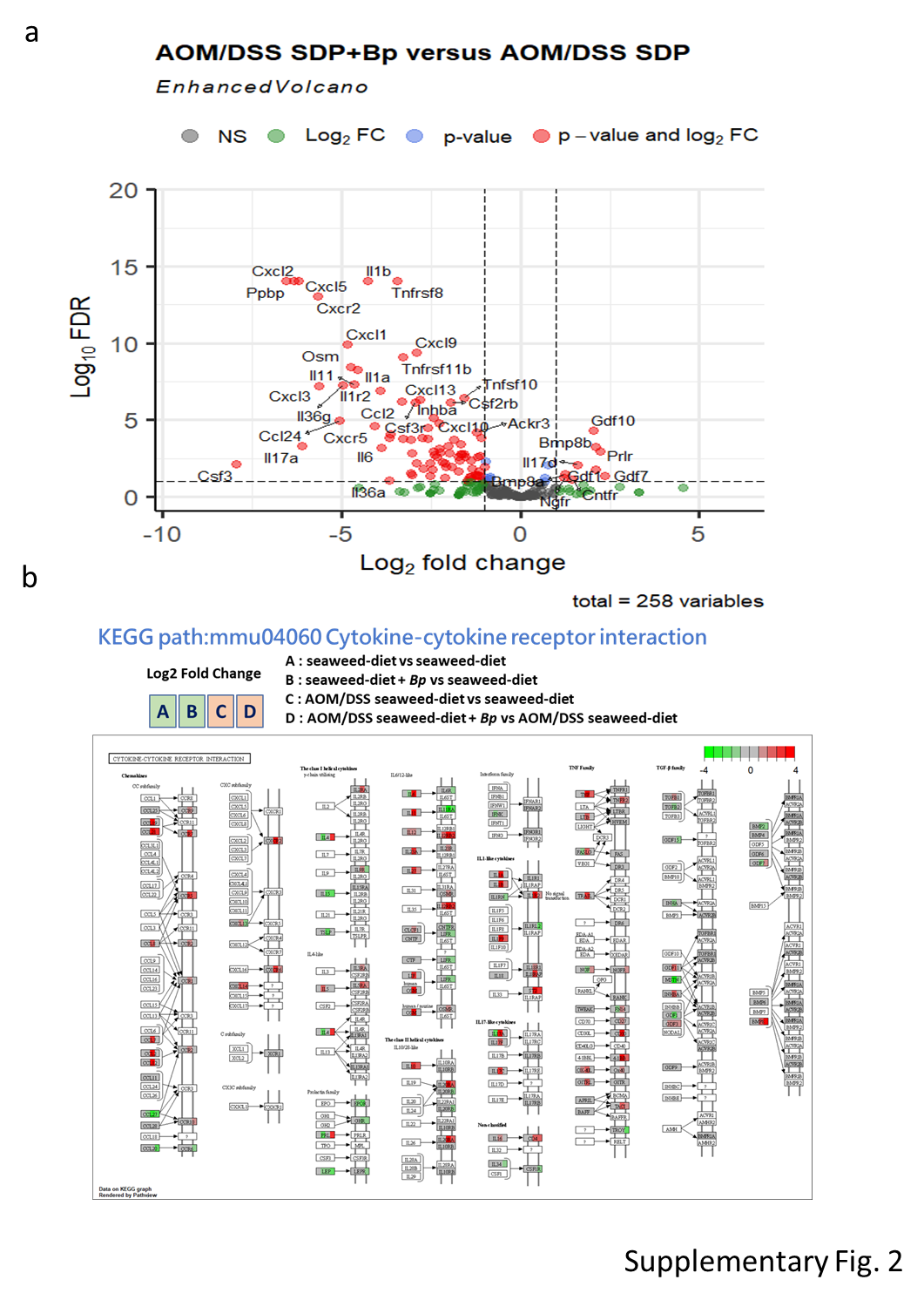


**Supplementary Figure 3.** Volcano plot (a) and KEGG pathway (b) analyses of cytokine-cytokine receptor interaction pathway (KEGG: mmu04060).


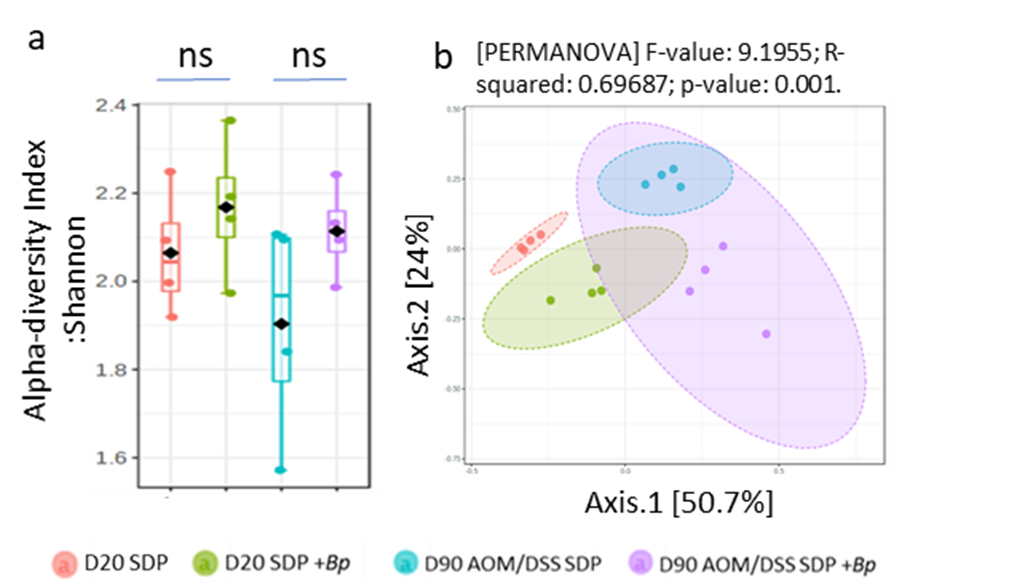


**Supplementary Figure 4.** (a) Shannon diversity index was computed to evaluate the richness and evenness of the microbial community in AOM/DSS-treated mice with seaweed diet or seaweed diet + B. plebeius colonization. (b) Beta diversity was computed using the Bray-Curtis index and PCoA plot. The boxes or circles in red and green represent the controls, and the boxes or circles in blue and purple represent AOM/DSS-treated mice with seaweed diet and seaweed diet + B. plebeius treatment, respectively. Also, the statistical significance of the clustering pattern in ordination plots can be evaluated using anyone among Permutational ANOVA (PERMANOVA). Beta diversity analysis is performed using the phyloseq package.

a

b

**Supplementary Figure 5. Fecal metabolite analyses of seaweed diet + *E. coli* colonization in gnotobiotic mice.** (a) Fecal short-chain fatty acid levels were quantified using gas chromatography-mass spectrometry, and the levels of acetic, propionic, isobutyric, butyric, isovaleric, valeric, hexanoic, heptanoic, octanoic, and decanoic acids were calculated as um of fecal samples. (b) Fecal bile acids were analyzed using ultra-high-performance liquid chromatography-parallel reaction monitoring with tandem mass spectrometry.


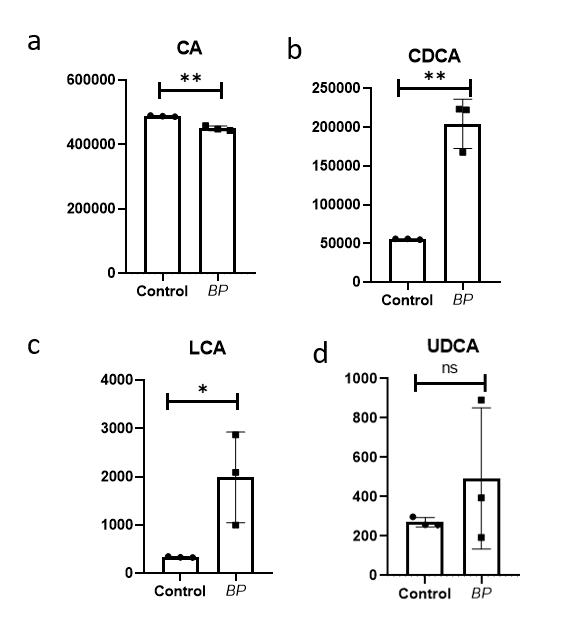


**Supplementary Figure 6. In vitro culture of Bp with primary acids.** In our study, we cultured anaerobic Bp in vitro and supplemented the culture with primary bile acids. The incubation period lasted for five days under anaerobic conditions to allow for the metabolism of the primary bile acids by the bacteria. After the incubation period, we analyzed the concentration of secondary bile acids produced during this time. The concentration of CA, CDCA, LCA and UDCA in bacteria culture medium were analyzed using ultra-high-performance liquid chromatography-parallel reaction monitoring with tandem mass spectrometry. *, P<0.5; **, P<0.01, ns: not significant.


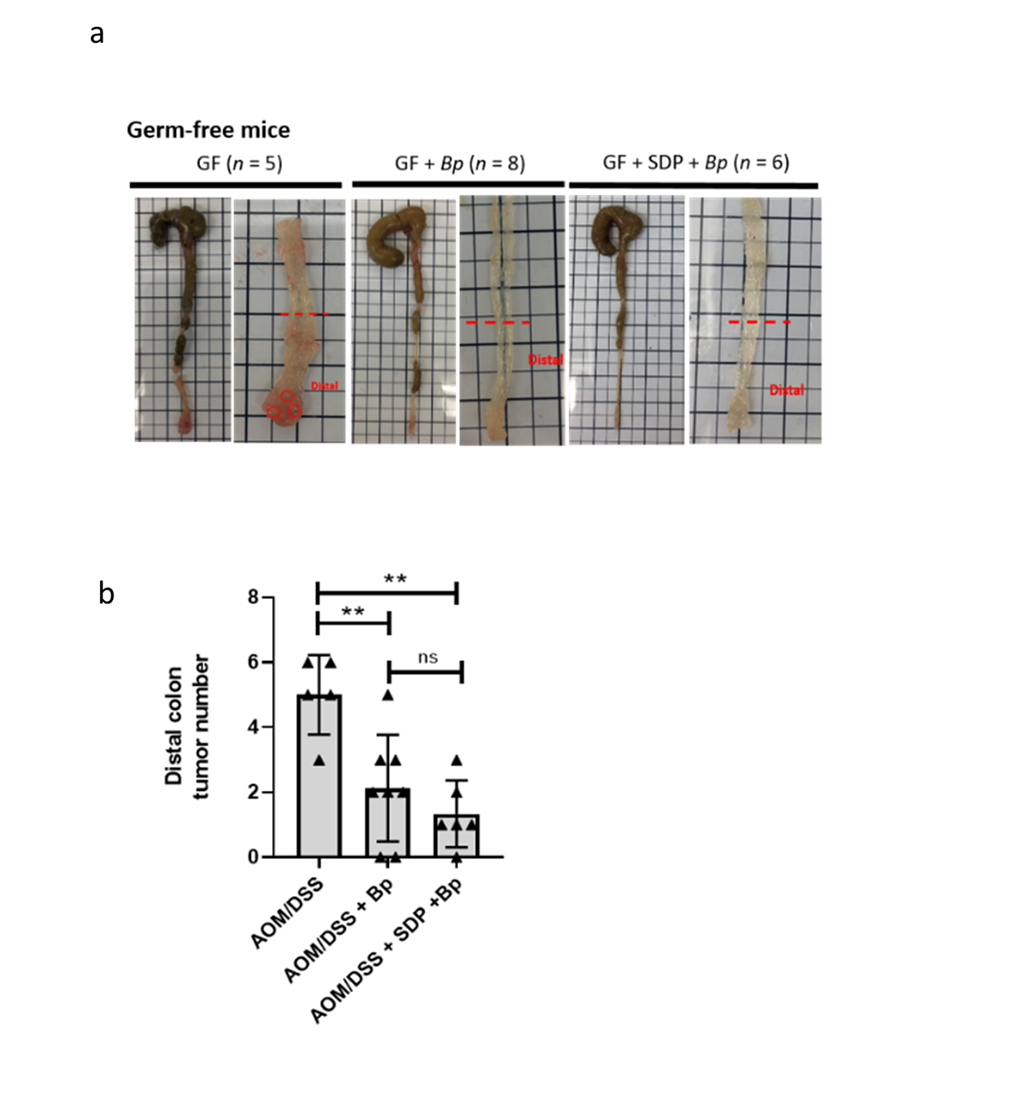


**Supplementary Figure 7.** The Tumor number measurement of seaweed diet (SDP) and *B. plebeius* colonized gnotobiotic mice treated with AOM/DSS induced inflammatory carcinogenesis. (a) Colon tissues were harvested from AOM/DSS-treated germ-free mice of control (n = 5), *B. plebeius* only (n = 8) and seaweed diet plus B. plebeius (n = 6). (b) Distal colon tumor number of AOM/DSS treated germ-free mice were calculated by the quantification of the colon tissue tumor numbers. **P < 0.01 and ns: not significant.

b.

**Supplementary Figure 8. Fecal metabolite analyses of seaweed diet + *B. plebeius* colonization in SPF mice.** Fecal short-chain fatty acid levels were quantified using gas chromatography-mass spectrometry, and the levels of acetic, propionic, isobutyric, butyric, isovaleric, valeric, hexanoic, heptanoic, octanoic, and decanoic acids were calculated as um of fecal samples. (b) Fecal bile acids were analyzed using ultra-high-performance liquid chromatography-parallel reaction monitoring with tandem mass spectrometry.


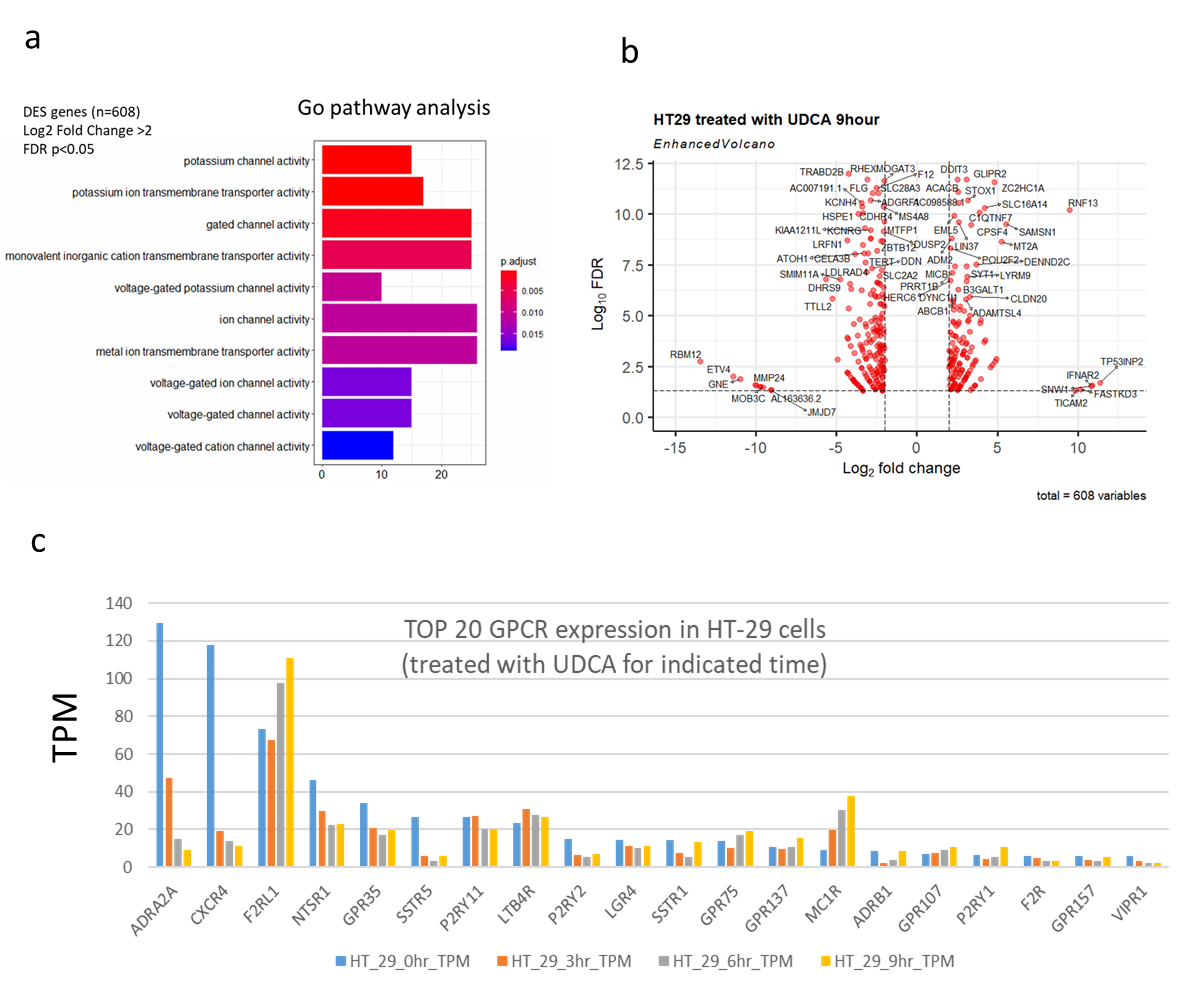


**Supplementary Figure 9.** RNAseq analysis of HT-29 cells treated with 500uM UDCA for 3, 6 and 9 hours compared with 0 hour (control). (a) Go pathway analysis of the differentially expressed genes (Log2 Fold Change >2 and FDR p<0.05). (b) Volcano plot analysis of the differentially expressed genes (n=608). (c) The TPM of top 20 GPCR expression in HT-29 Cells.

**Supplementary Table1**. Fecal bile acids were analyzed using ultra-high-performance liquid chromatography coupled with tandem mass spectrometry. Eighteen bile acids were detected among the 41 targeted bile acids examined. Each group has three mice and the value is mean ± standard deviation (nmole/L).


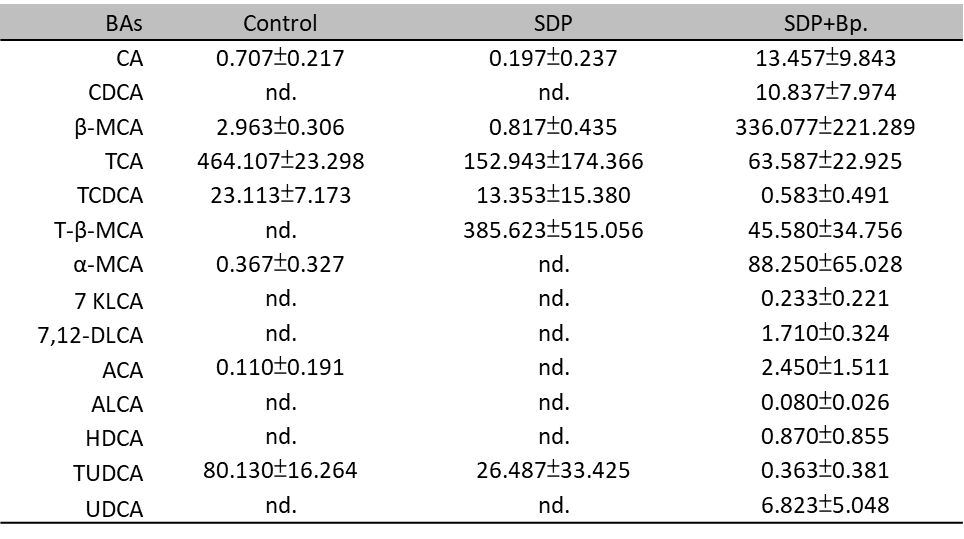


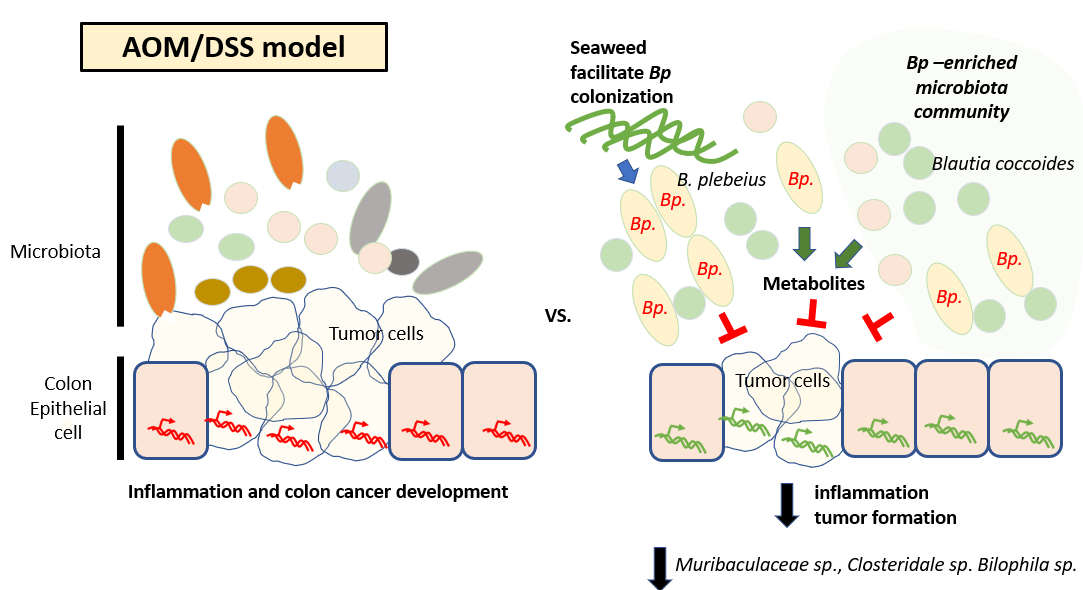


**Supplementary Figure 10. The complex interplay of human gut microbe *Bacteroides plebeius* (*B. plebeius*), colonization suppresses colon tumor development induced by azoxymethane (AOM) and dextran sulfate sodium (DSS).** This tumor suppression correlates with a decrease in the abundance of microbial families, such as *Muribaculaceae sp*., *Clostridiales sp.*, and *Bilophila sp*. Additionally, colonization by *B. plebeius* in gnotobiotic mice enhances the production of beneficial metabolites, including propionic acid, taurocholic acid, cholic acid, alpha- and beta-muricholic acids, and ursodeoxycholic acid. These metabolites are recognized for their anti-inflammatory and tumor-suppressive effects. This schematic diagram illustrated that *B. plebeius* can effectively restructure the gut microbial community and produce beneficial metabolites, leading to the inhibition of colitis-associated colon cancer development.
